# Rhus Toxicodendron—(Poison Oak)

**Published:** 1882-02-20

**Authors:** R. L. Hinton

**Affiliations:** Arkansas


### RHUS TOXICODENDRON—(POISON OAK.)

BY R. L. HINTON, M. D., OF ARKANSAS.

Having noticed quite a number of remedies offered in the Journals during the past twelve months for the cure of the distressing rash caused by poison oak, I offer a specific in "sassafras tea"—an infusion of the root, or better, the bark of the root of red sassafras—for there are certainly two varieties, known as the red and the white sassafras. Bathe diseased surface frequently, or constantly in the cold infusion, or better, apply a wet cloth. I am very susceptible of this poison myself, and stumbled upon this remedy from painful experience about fifteen years ago. I have used no other remedy since that time, though frequently called upon to prescribe for this poison, and in several cases that were aggravated by the application of various remedies, many of them worse than the disease. In such cases I advise the warm infusion as a drink three times a day, which, with sugar and cream, makes a pretty good substitute for tea or coffee. I have found this remedy a success in other skin diseases.

